# Very high HDL-C (high-density lipoprotein cholesterol) is associated with increased cardiovascular risk in patients with NSTEMI (non-ST-segment elevation myocardial infarction) undergoing PCI (percutaneous coronary intervention)

**DOI:** 10.1186/s12872-023-03383-9

**Published:** 2023-07-17

**Authors:** Lijuan Chen, Yuanyuan Zhao, Zheng Wang, Yifei Wang, Xiangwei Bo, Xiaoxi Jiang, Chunshu Hao, Chengwei Ju, Yangyang Qu, Hongjian Dong

**Affiliations:** 1 Department of Cardiology, Zhongda Hospital, Southeast University, 210009 Nanjing, China; 2 Department of Cardiology, Nanjing Lishui People’s Hospital, Zhongda Hospital Lishui Branch, 211200 Nanjing, China; 3 School of Medicine, Southeast University, 210009 Nanjing, China

**Keywords:** Cholesterol, High-density lipoprotein, Dyslipidemia, Percutaneous coronary intervention, MACCE, NSTEMI

## Abstract

**Background:**

Studies in populations with or without cardiovascular disease have shown that very high HDL-C levels are associated with an increased risk of cardiovascular events. However, the exact relationship between HDL-C levels and long-term prognosis remains unknown in patients with myocardial infarction (MI) undergoing percutaneous coronary intervention (PCI).

**Methods:**

This was a post hoc secondary analysis of long-term follow-up results in patients undergoing PCI open-label, observational cohort study. Patients with MI who had undergone PCI were enrolled. Restricted cubic spline (RCS) analysis and logistic regression analysis were performed to assess the relationship between HDL-C levels and the risk of cardiovascular events.

**Results:**

A total of 1934 patients with MI undergoing PCI were enrolled in our analysis and our population was divided in 3 groups according to the HDL-C plasma levels: HDL-C < 40 mg/dL (low HDL-C); HDL-C between 40 and 80 mg/ dL (medium HDL-C); and HDL-C > 80 mg/dL (high HDL-C). RCS analysis showed a nonlinear U-shaped association between HDL-C levels and major adverse cardiac and cerebrovascular events (MACCE) in patients with NSTEMI with adjusted variables. After adjusting for potential confounders, the follow-up analysis indicated that high risk group had elevated occurrence of MACCE than low risk group (HDL-C 35 and 55 mg/dL) (OR:1.645, P = 0.006).

**Conclusions:**

Our analysis demonstrated that there is a U-shaped association between HDL-C and MACCE in patients with NSTEMI undergoing PCI.

**Supplementary Information:**

The online version contains supplementary material available at 10.1186/s12872-023-03383-9.

## Introduction

High-density lipoprotein cholesterol (HDL-C) has been considered for many years to be an atherosclerotic and cardioprotective property, a postulate mainly based on seminal epidemiological studies indicating that each 1 mg/dL increase in HDL-C is accompanied by a ≈ 2–3% reduction in the risk of cardiovascular death [1–7]. However, recent clinical trials and genetic studies investigating the rise of HDL-C through pharmacological therapy or genetic polymorphisms, respectively, have failed to show an effect on major adverse cardiac and cerebrovascular events (MACCE) [8–11]. These findings challenge the therapeutic value of pharmacological HDL-C elevating treatment and contribute to in-depth studies of prognostic value of HDL-C [12–14]. More recently, studies in populations free of cardiovascular disease have shown that very high HDL-C levels are associated with an increased mortality risk, and the same conclusion has been confirmed in population with coronary artery disease or hypertension [7, 15–18]. Nevertheless, the exact relationship between HDL-C levels and specific cardiovascular events remains unknown, especially in a high-risk population like patients with myocardial infarction (MI) undergoing percutaneous coronary intervention (PCI).

To further clarify this issue, we have analyzed the relationship between HDL-C levels and long-term prognosis in patients MI undergoing PCI in a high-volume PCI centre, with a long-term follow-up.

## Methods

### Study population

This was a post hoc secondary analysis of an open-label, observational cohort study in which we enrolled patients undergoing PCI [18, 19]. The study was conducted in consecutive patients enrolled at a single high-volume PCI center between July 2009 and August 2011. Qualitative and quantitative coronary angiographic analyses were performed according to standard methods. PCI was performed using standard techniques. All patients received loading doses of aspirin (300 mg) and clopidogrel (300 mg) before coronary intervention unless they were already receiving antiplatelet therapy. The treatment strategy, stenting techniques, selection of stent type, and use of glycoprotein IIb/IIIa receptor inhibitors or intravascular ultrasound were all left to the surgeon’s discretion. All patients were prescribed 100 mg/day aspirin indefinitely and clopidogrel 75 mg/day for at least the first 12 months after the procedure [18]. In brief, we included men and women over 18 years old who underwent PCI and had HDL-C levels measured during hospitalization. For our analysis, patients undergoing PCI were selected on the basis of the following inclusion criteria: age ≥ 18 years; confirmed diagnosis of MI; at least one follow-up visit. We excluded patients without record of HDL-C level. The trial was conducted according to the Declaration of Helsinki and was approved by the ethics committee of the First Affiliated Hospital of Zhengzhou University.

### Cardiovascular risk factor and end points

Information on demographics and relevant risk factors was obtained at enrollment, including age, sex, stroke history, old myocardial infarction (OMI), heart failure, atrial fibrillation, peripheral vascular disease, hypertension, diabetes, and smoking habit. Auxiliary examination information was obtained during hospitalization including HDL-C, low-density lipoprotein cholesterol (LDL-C), total cholesterol (TC), triglycerides (TG), glucose, uric acid (UA) and serum creatinine (Scr).

Primary end points were defined as major adverse cardiac and cerebrovascular events (MACCE), namely death, MI and stroke. Clinical follow-up was conducted through patient visits, telephone interviews, and review of medical records. Independent investigators entered the data, and an independent committee adjudicated clinical events. Between July 2009 and August 2011, 2 735 patients were treated by PCI [18]. 2533 patients (92.6%) were followed up for a median of 29.8 months (interquartile 25.6–34 months).

### Statistical analysis

Data are presented as mean (SD) for continuous variables and in percentage form for categorical variables. ANOVA and χ2 distribution were used for exploratory statistics. Our study population was divided into three groups based on HDL-C levels (mg/dL < 40, 40–80, and > 80) to examine the association between HDL-C levels and the incidence of cardiovascular events. Restricted cubic spline analysis was performed to assess the relationship between HDL-C levels and the risk of cardiovascular events. A logistic regression analysis was applied to assess the effects of HDL-C on cardiovascular events during follow-up after adjusting for age, sex, hypertension, diabetes, baseline BP values, baseline heart rate, smoking habit, LDL, TG, TC, UA and Scr. A 2-tailed P < 0.05 was considered statistically significant. Statistical analysis was performed using IBM SPSS 25 (IBM Corporation, Armonk, NY) and R Statistical Software (version 4.1.0; R Foundation for Statistical Computing).

## Results

**Fig. 1:**
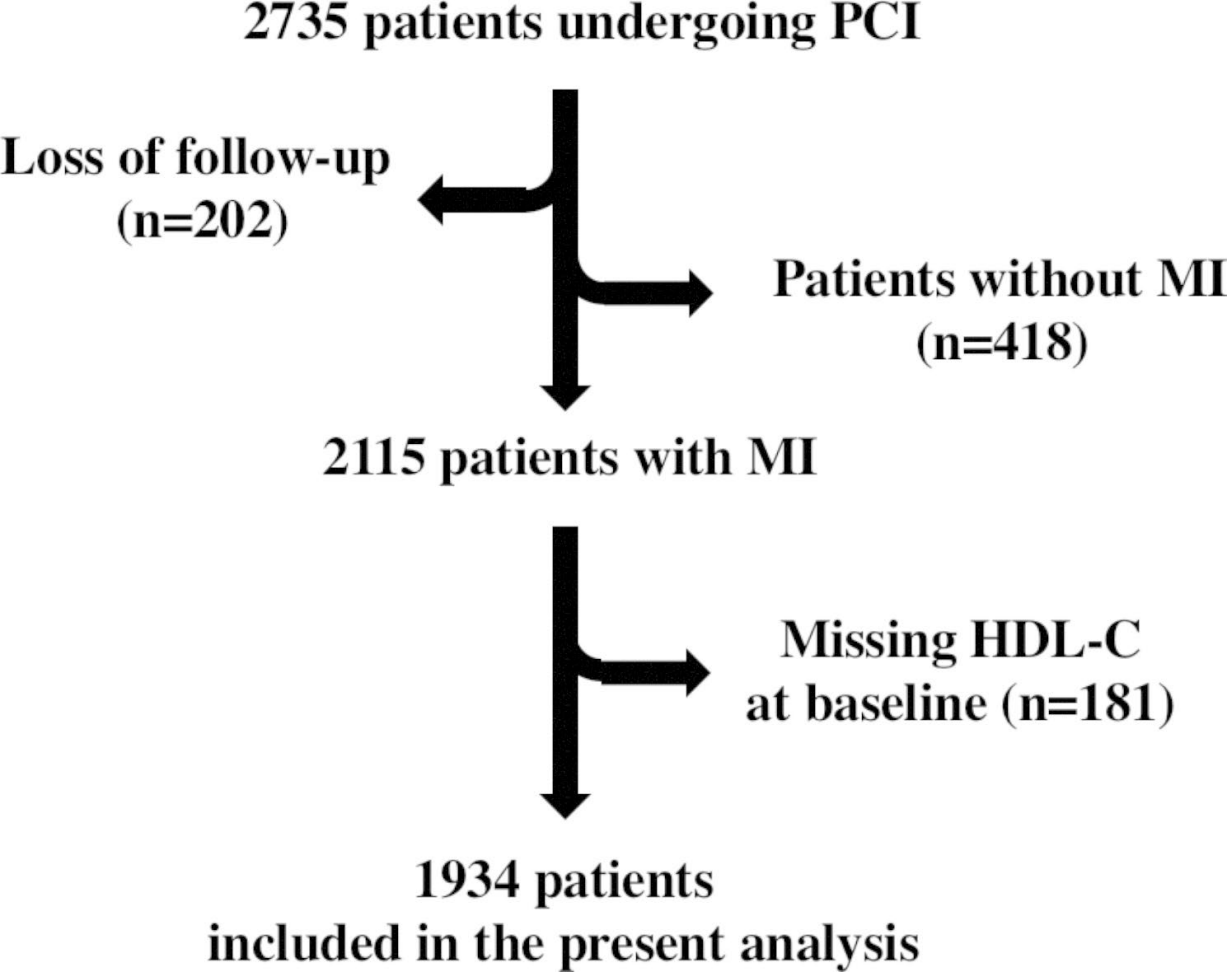
Flow chart of the study. Abbreviations: PCI, Percutaneous coronary intervention; HDL-C, High-density lipoprotein cholesterol; MI, Myocardial infarction

A total of 2 533 patients were enrolled at the high-volume PCI centre; of these, 1934 patients (1305 men [67.5%]; 629 women [32.5%]) were included in our analysis (Fig. 1). Patients with ST-elevation myocardial infarction (STEMI) accounted for 29.3% of cases and patients with non-ST elevation ACS(NSTE-ACS) accounted for 70.7% of cases. Our population was divided in 3 groups (Table 1) according to the HDL-C plasma levels: specifically, the first group includes 1011 patients with HDL-C below 40 mg/dL (low HDL-C); the second group includes 905 patients with HDL-C plasma levels between 40 and ≤ 80 mg/dL (medium HDL-C); and 18 patients with HDL- C > 80 mg/dL constituted the third group (high HDL-C). The main demographic and clinical characteristics of these 3 groups are displayed in Table 1. Importantly, no significant difference among our groups was found in terms of medications (Table 2).

**Table 1:** Baseline characteristics of different levels of HDL-C.

Parameter	Low HDL cholesterol n = 1011	Medium HDL cholesterol n = 905	High HDL cholesterol n = 18	P Value
Age, yrs	58.68(11.34)	60.90(10.76)	63.72(11.28)	0.001
Female, n (%)	245(24.2)	378(41.8)	6(33.3)	0.001
Heart failure, n (%)	118(11.7)	113(12.5)	4(22.0)	0.362
Atrial fibrillation, n (%)	9(0.9)	11(1.2)	0	0.711
OMI, n (%)	50(4.9)	32(3.5)	0(4.8)	0.208
Stroke, n (%)	47(4.6)	48(5.3)	0	0.502
Peripheral vascular disease, n (%)	3(0.3)	0	0	0.254
Hypertension, n (%)	511(50.5)	485(51.4)	11(61.1)	0.642
Diabetes mellitus, n (%)	243(24.0)	174(19.2)	3(16.7)	0.035
SBP, mm Hg	105.16(28.81)	98.32(27.66)	102.94(27.66)	0.001
DBP, mm Hg	77.54(11.53)	76.15(12.48)	76.44(13.18)	0.042
Heart rate, bpm	73.31(11.45)	71.04(11.96)	68.85(12.60)	0.001
HDL-cholesterol, mg/dL	32.40(5.09)	49.72(7.97)	97.23(17.43)	0.001
LDL-cholesterol, mg/dL	96.27(35.87)	107.72(38.82)	96.33(45.50)	0.001
Total cholesterol, mg/dL	72.75(18.50)	81.52(18.76)	80.20(21.99)	0.001
Triglycerides, mg/dL	189.49(143.4)	150.01(86.24)	138.21(75.62)	0.001
Current smokers, n (%)	375(37.1)	267(29.5)	5(27.8)	0.002
Glucose, mg/dL	109.87(55.45)	108.49(61.12)	102.13(37.52)	0.767
Serum creatinine, mg/dL	0.85(0.39)	0.79(0.38)	0.84(0.39)	0.002
Uric acid, mg/dL	52.88(15.64)	48.58(15.51)	37.14(13.85)	0.001
Total chronic occlusions, n (%)	76(7.5)	84(9.3)	2(11.1)	0.347
Location of target lesions, n (%)				
LM	32(3.2)	29(3.3)	0	0.743
LAD	830(82.1)	740(81.8)	14(77.8)	0.885
LCX	486(48.1)	439(48.5)	13(72.2)	0.127
RCA	506(50.0)	440(48.6)	7(38.9)	0.556
Number of stents per patient	2.15(1.23)	2.12(1.26)	2.17(1.25)	0.847
Total stent length per patient	50.04(31.66)	48.44(32.27)	52.39(36.05)	0.507
Number of treated vessels	1.52(0.66)	1.50(0.65)	2.12(1.26)	0.834

Abbreviations: HDL-C, High-density lipoprotein cholesterol; OMI, Old myocardial infarction; SBP, Systolic blood pressure; DBP, Diastolic blood pressure; LDL-C, Low-density lipoprotein cholesterol; LAD, Left anterior descending artery; LCX, Left circumflex artery; NSTE-ACS, Non-ST elevation acute coronary syndromes; PCI, Percutaneous coronary intervention; RCA, Right coronary artery

**Table 2:** Distribution of Medications in Our Population

Parameter	Low HDL cholesterol n = 1011	Medium HDL cholesterol n = 905	High HDL cholesterol n = 18	P Value
ACE inhibitor or ARNI, n (%)	582(42.4)	482(46.7)	10(44.4)	0.166
Statins, n (%)	1011(93.9)	852(94.1)	18(100)	0.545
β-Blockers, n (%)	723(71.5)	644(71.2)	13(72.2)	0.982
Calcium channel blockers, n (%)	233(23.0)	238(26.3)	6(33.3)	0.178
Aspirin, n (%)	1000(98.9)	891(98.6)	18(100)	0.703
Clopidogrel, n (%)	976(96.6)	865(95.6)	18(100)	0.669

Abbreviations: HDL-C, High-density lipoprotein cholesterol; ACEI, Angiotensin-converting enzyme inhibitor; ARNI, Angiotensin receptor neprilysin inhibitor

**Fig. 2:**
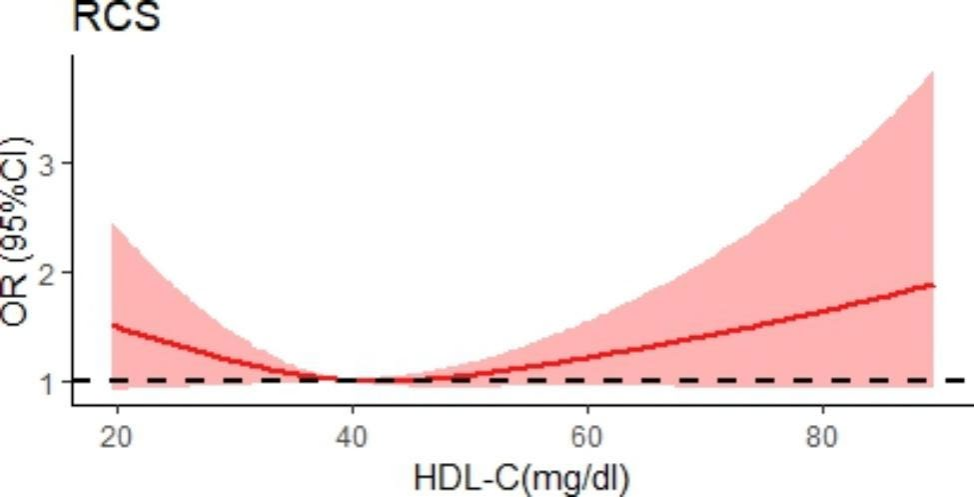
Spline curves of the Logistics regression models for HDL-C and the risk of cardiovascular events in patients with MI undergoing PCI. Spline plot showing the association between HDL-C and risk of cardiovascular events in patients with MI undergoing PCI. The shaded area represents the 95% CI. Abbreviations: PCI, Percutaneous coronary intervention; HDL-C, High-density lipoprotein cholesterol; MI, myocardial infraction; MACCE, Major adverse cardiac and cerebrovascular events; RCS, Restricted cubic spline; CI, Confidence interval; OR, Odds ratio

**Fig. 3:**
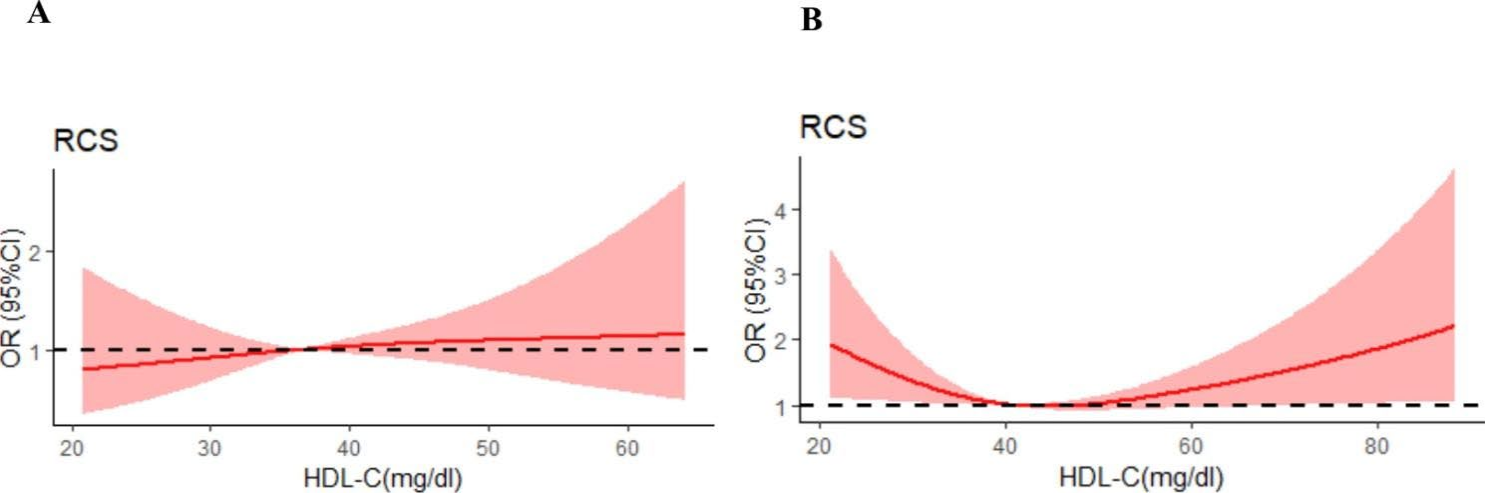
Spline curves of the Logistics regression models for HDL-C and the risk of cardiovascular events in patients with STEMI and patients with NSTEMI undergoing PCI. Spline plots showing the association between HDL-C and risk of cardiovascular events in patients with STEMI (A) and patients with NSTEMI (B) undergoing PCI. The shaded area represents the 95% CI. Abbreviations: PCI, Percutaneous coronary intervention; HDL-C, High-density lipoprotein cholesterol; MI, Myocardial infraction; STEMI, ST-segment elevation myocardial infarction; NSTEMI, Non-ST-segment elevation myocardial infarction; MACCE, Major adverse cardiac and cerebrovascular events; RCS, Restricted cubic spline; CI, Confidence interval; OR, Odds ratio

We then plotted the spline curves of the Logistics regression models to estimate the relative hazard ratio in our population, no remarkable nonlinear U-shaped association between HDL-C levels and MACCE was not observed in patients with MI(Fig. 2). Intriguingly, when subdividing our population in patients with STEMI and patients with NSTEMI, we found a nonlinear U-shaped association between HDL-C levels and MACCE in patients with NSTEMI (P = 0.026) (Fig. 3A and B).

**Fig. 4:**
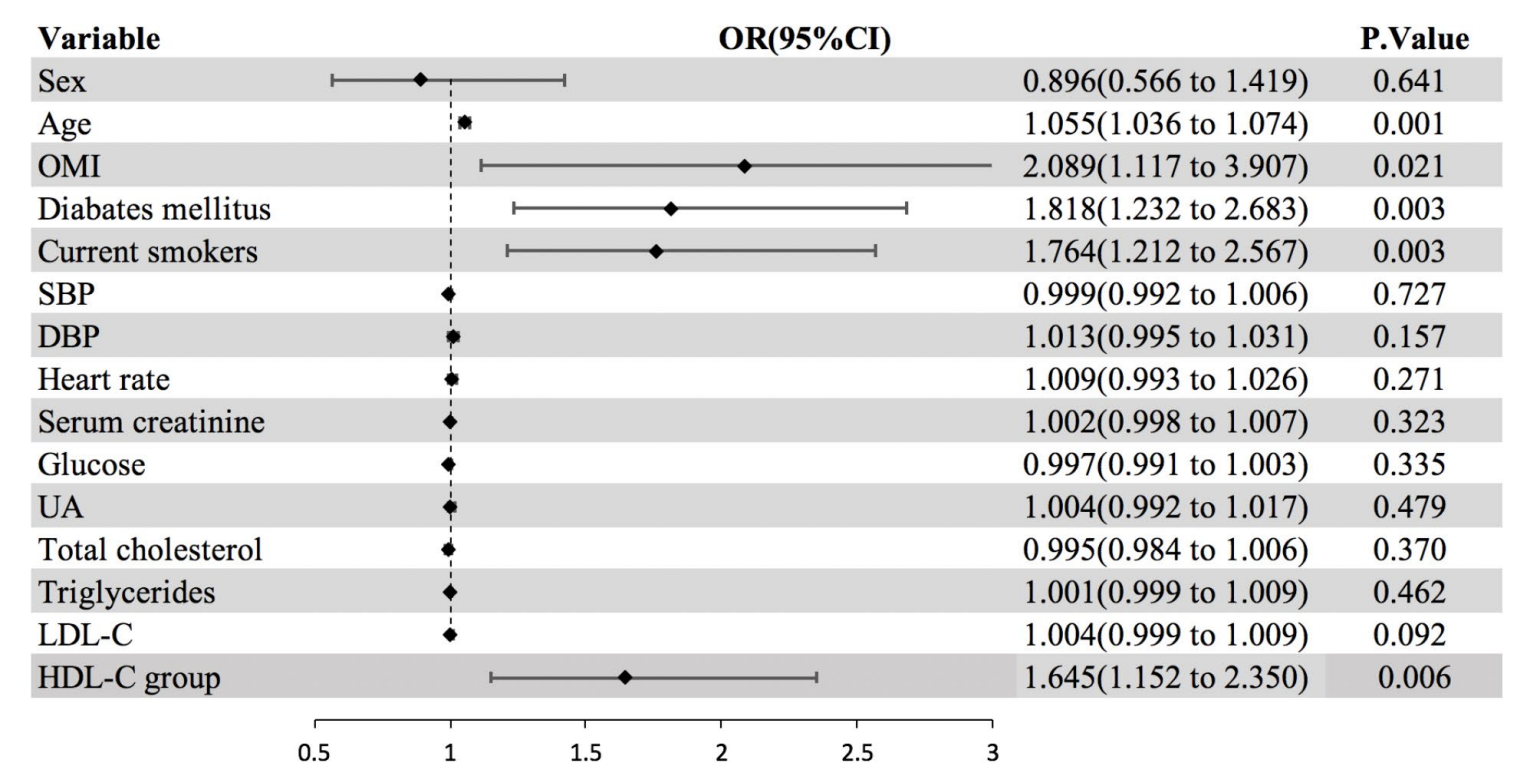
The follow-up analysis of MACCE. Patients were divided into high-risk group(< 35 and > 55 mg/dl) and low-risk group (35–55 mg/dl) according to their HDL-C levels. After having adjusted for potential confounders, the follow-up analysis indicated that high-risk group had elevated occurrence of MACCE than low-risk group in patients with NSTEMI (B) undergoing PCI (HR:1.723, P = 0.002). Abbreviations: OMI, Old myocardial infraction; SBP, Systolic blood pressure; DBP, Diastolic blood pressure; LDL-C, Low-density lipoprotein cholesterol; HDL-C group, High-density lipoprotein cholesterol group; NSTEMI, Non-ST-segment elevation myocardial infarction; OR, Odds ratio; CI, Confidence interval

Patients were divided into high-risk group (< 35 and > 55 mg/dl) and low-risk group (35–55 mg/dl) according to their HDL-C levels in patients with NSTEMI. As shown in Table 3, the incidences of death and MACCE were higher in the high-risk group. During the mean follow-up of 29 months, a total of 83 patients in high-risk group (n = 580) and 76 patients in low-risk group (n = 787) occurred MACCE. The incidence of MACCE in high-risk group were higher than that in the low-risk group (14.30% vs. 9.7%, P = 0.005). We then compared baseline difference in patients with NSTEMI (Additional File Table 1). As shown in Fig. 4, after having adjusted for potential confounders, the follow-up analysis indicates that high-risk group had elevated occurrence of MACCE than low-risk group in patients with NSTEMI (OR:1.645, P = 0.006).

**Table 3:** Clinical events according to the levels of HDL-C in patients with NSTEMI

Level of HDL-C, mg/dL	< 35 n = 397	35–55 n = 787	> 55 n = 183	P Value
MACCE, n (%)	58(14.6)	76(9.7)	25(13.7)	0.028
All-cause death, n (%)	40(10.1)	45(5.7)	14(7.7)	0.023
MI, n (%)	18(4.5)	32(4.1)	9(4.9)	0.850
Stoke, n (%)	2(0.5)	10(1.3)	5(2.7)	0.079

Abbreviations: HDL-C, High-density lipoprotein cholesterol; MACCE, Major adverse cardiac and cerebrovascular events; MI, Myocardial infraction

## Discussion

Our results demonstrate that the association between HDL-C and risk of major adverse cardiac and cerebrovascular events (MACCE) is U-shaped in patients with NSTEMI undergoing PCI, with both low and high concentration groups appearing elevated occurrence of MACCE compared with the HDL-C 35 and 55 mg/dL group.

The mechanism of HDL protection for the heart mainly includes the reverse transport of cholesterol and its antioxidant capacity [20–26]. However, clinical trials and genetic studies investigating the rise of HDL-C using pharmacological therapy and genetic polymorphisms, respectively, have failed to show an effect on major adverse cardiac and cerebrovascular events (MACCE) [8–11]. Studies in population without cardiovascular disease have shown that extremely high HDL-C levels are associated with an increased risk of death and same results were found in people with coronary artery disease [15–17]. And very high Lp(a) levels (above the threshold of 60 mg/ dL) was associated with a higher recurrence of cardiovascular events In our study, the correlation of very high HDL and major adverse cardiac and cerebrovascular events (MACCE)were also observed in patients with NSTEMI undergoing PCI. The increased cardiovascular risk associated with elevated levels of HDL-C was not confirmed in patients with STEMI undergoing PCI. Erosion predominantly provokes NSTEMI while plaque rupture associates more commonly with STEMI. Lipid lowering, particularly statin therapy, lessens the lipid core, and augments the relative amount of fibrous tissue in atherosclerotic plaques [27] and 95% of our population undergone statin therapy. Another reason is the low proportion of events may result in less precise estimates compared with the overall cohort.

After having adjusted for potential confounders, we found high HDL-C concentration groups appear elevated occurrence of MACCE compared with the medium group in patients with NSTEMI undergoing PCI, which contradicts the protective effect of high HDL-C on the heart. Our observational study does not clarify whether the association between high HDL-C levels and increased cardiovascular risk is causal. Thus, we can only speculate on the possible pathogenetic mechanisms. A high HDL cholesterol reading cannot represent an efficient transport system moving large amounts of cholesterol to the liver for excretion, such as a problem with disembarkation at the liver. In fact, since cholesterol exchange between HDL particles and peripheral cells is bidirectional, it is possible that HDL could become a cholesterol donor to peripheral cells in the oversaturated state. Lipid trapped within HDL particles contribute to increased cardiovascular risk[28].

Immediate revascularization is the most effective intervention to reduce mortality after MI. Paradoxically, reperfusion of ischaemic tissue promotes further myocardial damage mediated by mitochondrial reactivation, rapid recovery of physiological pH, and excess ROS production. HDL has been suggested as a possible treatment strategy for patients with acute comorbid syndromes due to its antioxidant and pro-survival properties. Animal studies have confirmed that HDL isolated from healthy subjects protects the myocardium from ischemia/reperfusion injury. In contrast, HDL derived from patients in the acute phase of MI lost its ability to protect the heart from experimental MI [29]. These results may be related to serum amyloid A (SSA), whose level are positively correlated with cardiovascular disease [30–32]. SAA upregulation triggers its association with HDL, replacing ApoA-I as the main apolipoprotein of HDL [33, 34]. Binding of SAA to HDL helps to convert functional anti-atherosclerotic HDL to dysfunctional pro-atherosclerotic HDL. HDL particles containing SAA have a reduced ability to transport cholesterol in the reverse direction from macrophages. This correlates with the ability of SAA to interact with cell-surface proteoglycans, which prevents HDL from adequately interacting with the plasma membrane to promote cholesterol efflux [35, 36]. Another underlying mechanism is that there is an inverse U-shaped relationship between triglyceride-rich lipoprotein (TGRL) and HDL-C concentration. The ability of HDL to acquire cholesterol during lipolysis is reduced after the knockout of TGRL. Therefore, the transfer of free cholesterol to HDL via TGRL lipolysis may be the basis of the U-shaped relationship between HDL-C and cardiovascular diseases [37].

However, there are still several limitations in our study that can be improved in the future. Firstly, our observational study was from a single-center, therefore, our finding may not exactly apply to other population. The second limitation was the small number of patients with very high HDL-C; nevertheless, it did not undermine the demonstration of a significant increase in the cardiovascular risk after adjustment for confounders. The third limitation is that post-PCI management, such as lipid-lowering therapy, varied considerably compared to current clinical standards since the study period was over ten years ago. The fourth limitation is that we applied logistic regression analysis instead of COX proportional hazards model due to the lack of data about the time-event relationship. The fifth limitation is that the baseline information did not contain lipoprotein(a), which has been verified as an influencing factor of cardiovascular events in patients with AMI undergoing PCI [38, 39].

## Conclusion

Our data indicate that there is a U-shaped association between HDL-C and MACCE in patients with NSTEMI undergoing PCI during a long-term follow-up. Low and high concentration groups showed elevated risk of MACCE compared with the HDL-C 35 and 55 mg/dL group. Our finding may help physicians manage dyslipidemia in patients with NSTEMI undergoing PCI.

## Electronic supplementary material

Below is the link to the electronic supplementary material.

Additional File Table1: Baseline characteristics of different levels of HDL-C in patients with NSTEMI.

## Data Availability

The datasets used and analyzed during the current study are available from the corresponding author on reasonable request.

## Abbreviations

HDL: High-density lipoprotein
HDL-C: High-density lipoprotein cholesterol
PCI: Percutaneous coronary intervention
CHD: Coronary heart disease
MI: Myocardial infarction
STEMI: ST-segment elevation myocardial infarction
NSTEMI: Non-ST-segment elevation myocardial infarction
PCI: Percutaneous coronary intervention
MACCE: Major adverse cardiac and cerebrovascular events
TVR: Target vessel revascularization
OMI: Old myocardial infarction
SBP: Systolic blood pressure
DBP: Diastolic blood pressure
LDL-C: Low-density lipoprotein cholesterol
TC: Total cholesterol
TG: Triglycerides
UA: Uric acid
Scr: Serum creatinine
ACEI: Angiotensin-converting enzyme inhibitor
ARNI: Angiotensin receptor neprilysin inhibitor
CETP: Cholesteryl ester transfer protein
AF: Atrial fibrillation
TGRL: Triglyceride-rich lipoprotein
SSA: Serum amyloid A
RCS: Restricted cubic spline
OR: Odds ratio
CI: Confidence interval
