# Supplementary material for: Very high HDL-C (high-density lipoprotein cholesterol) is associated with increased cardiovascular risk in patients with NSTEMI (non-ST-segment elevation myocardial infarction) undergoing PCI (percutaneous coronary intervention)
Source: BMC Cardiovasc Disord. 2023 Jul 17;23:357. doi: 10.1186/s12872-023-03383-9 (PMC10353083; doi:10.1186/s12872-023-03383-9)
Supplement: Supplementary file 1 — Additional File Table1: Baseline characteristics of different levels of HDL-C in patients with NSTEMI. [file 12872_2023_3383_MOESM1_ESM.docx]

Supplement Table 1 Baseline characteristics of different levels of HDL-C in patients with NSTEMI.

| **Level of HDL-C, mg/dL** | **<35**  **n=397** | **35-55**  **n=787** | **>55**  **n=183** | **P Value** |
| --- | --- | --- | --- | --- |
| Age, yrs | 59.11(10.74) | 60.74(10.74) | 62.67(10.97) | 0.001 |
| Female, n (%) | 91(22.9) | 311(39.5) | 96(52.5) | 0.001 |
| Heart failure, n (%) | 50(12.6) | 90(11.5) | 27(14.8) | 0.462 |
| Atrial fibrillation, n (%) | 6(1.5) | 8(1.0) | 2(1.1) | 0.752 |
| OMI, n (%) | 31(7.8) | 31(3.9) | 5(2.7) | 0.005 |
| Stroke, n (%) | 16(4.0) | 38(4.8) | 9(4.9) | 0.807 |
| Peripheral vascular disease, n (%) | 1(0.3) | 1(0.1) | 0 | 0.744 |
| Hypertension, n (%) | 213(53.7) | 449(57.1) | 90(49.5) | 0.143 |
| Diabetes mellitus, n (%) | 104(26.2) | 170(21.6) | 26(14.2) | 0.005 |
| SBP, mm Hg | 108.16(29.27) | 103.34(27.90) | 93.85(26.12) | 0.001 |
| DBP, mm Hg | 78.60(11.45) | 76.85(11.43) | 74.03(11.91) | 0.001 |
| Heart rate, bpm | 73.72(11.54) | 72.44(11.08) | 66.94(10.29) | 0.001 |
| HDL-cholesterol, mg/dL | 29.63(3.81) | 43.16(5.26) | 65.04(12.94) | 0.001 |
| LDL-cholesterol, mg/dL | 93.35(32.60) | 106.78(36.32) | 114.93(40.94) | 0.001 |
| Total cholesterol, mg/dL | 151.84(38.80) | 170.26(40.28) | 181.06(44.41) | 0.001 |
| Triglycerides, mg/dL | 214.23(185.9) | 166.50(100.9) | 136.65(76.86) | 0.001 |
| Current smokers, n (%) | 151(38.0) | 230(29.2) | 37(20.2) | 0.001 |
| Glucose, mg/dL | 104.63(35.98) | 104.75(37.45) | 96.54(28.22) | 0.018 |
| Serum creatinine, mg/dL | 0.85(0.46) | 0.79(0.37) | 0.79 (0.29) | 0.026 |
| Uric acid, mg/dL | 53.38(15.37) | 49.96(14.43) | 47.18(15.37) | 0.001 |
| Total chronic occlusions， n (%) | 40(10.1) | 62(7.9) | 19(10.4) | 0.334 |
| Location of target lesions, n (%) | | | | |
| LM | 17(4.3) | 21(2.7) | 7(3.8) | 0.309 |
| LAD | 322(81.1) | 624(79.3) | 141(77.0) | 0.515 |
| LCX | 199(50.1) | 379(48.2) | 92(50.3) | 0.762 |
| RCA | 196(49.4) | 390(50.1) | 78(42.6) | 0.223 |
| Number of stents per patient | 2.13(1.21) | 2.13(1.25) | 1.96(1.23) | 0.233 |
| Total stent length per patient | 49.30(31.54) | 48.93(32.49) | 45.48(32.14) | 0.369 |
| Number of treated vessels | 1.52(0.65) | 1.49(0.65) | 1.48(0.66) | 0.633 |

Abbreviations: HDL-C, High-density lipoprotein cholesterol; OMI, Old myocardial infarction; SBP, Systolic blood pressure; DBP, Diastolic blood pressure; LDL-C, Low-density lipoprotein cholesterol; LAD, Left anterior descending artery; LCX, Left circumflex artery; NSTE-ACS, Non-ST elevation acute coronary syndromes; PCI, Percutaneous coronary intervention; RCA, Right coronary artery.
